# Paeoniflorin attenuates DHEA-induced polycystic ovary syndrome via inactivation of TGF-β1/Smads signaling pathway *in vivo*

**DOI:** 10.18632/aging.202564

**Published:** 2021-02-26

**Authors:** Jie Zhou, Yong Tan, Xudong Wang, Meihong Zhu

**Affiliations:** 1 Nanjing University of Chinese Medicine, Nan Jing 210023, PR China; 2 Department of Chinese Medicine, The First People's Hospital of Nantong, Nan Tong 226001, Jiangsu, PR China; 3 Department of Reproductive Medicine, Affiliated Hospital of Nanjing University of Chinese Medicine, Nanjing 210029, Jiangsu, PR China; 4 Department of Pharmacy, The First People's Hospital of Nantong, Nan Tong 226001, Jiangsu, PR China

**Keywords:** paeoniflorin, ovarian fibrosis, polycystic ovary syndrome, TGF-β1/Smads signaling pathway

## Abstract

Polycystic ovarian syndrome (PCOS) is one of the most common reproductive endocrine disorders which are involved in complicated and unknown pathogenic mechanisms. Paeoniflorin (PAE) plays a significant anti-fibrotic role according to previous studies. The aim of the present study was to investigate the effect of PAE on ovarian fibrosis and its underlying mechanism in PCOS development. An animal model of PCOS was established by subcutaneous injection of 60mg/kg/d dehydroepiandrosterone (DHEA) for 35 consecutive days. Rats in PAE-L, PAE-M and PAE-H groups were administrated by gavage with PAE (20, 40, 80 mg/kg/d) for 4 weeks. Our results indicated that DHEA-induced PCOS rats showed similar phenotypes with PCOS patients. PAE could significantly block the DHEA-induced decline of ovary weight and organ coefficient, shorten the prolonged diestrus period, and regulate the irregular estrous cycle of PCOS rats. Moreover, PAE regulated reproductive hormone levels and improved ovarian fibrosis induced by DHEA. PAE treatment could also reduce the expression levels of TGF-β1 and Smad3, and increase the expression levels of Smad7 and MMP2. In conclusion, PAE significantly attenuated the ovarian fibrosis in PCOS, which could be mediated by TGF-β1/Smads signaling pathway. Herein, PAE can be used for the treatment of ovarian fibrosis in PCOS progression.

## INTRODUCTION

Polycystic ovary syndrome (PCOS) is recognized as the one of the most common reproductive endocrine diseases in reproductive women with an incidence rate of 5–20% worldwide [1]. It is characterized by oligo-ovulation, menstrual irregularities, hyperandrogenism, and the occurrence of polycystic ovaries [2, 3]. According to recent studies, the risk factors for the complications of PCOS are remarkably increasing, such as infertility, type 2 diabetes mellitus, endometrial cancer, obesity, psychosocial issues and cardiovascular diseases [3–5].

The pathogenic mechanisms of PCOS are intricate and remain unclear, which may be related to neuroendocrine axis dysfunction, hyperandrogenemia, insulin resistance, ovarian dysfunction, disorders of genetics and ovarian fibrosis [6–8]. Ovarian fibrosis, one of the significant pathological factors in ovarian diseases, hasn’t attracted much attention although it frequently triggers ovarian dysfunction [9]. The notion of ovarian fibrosis in PCOS was first brought up by Hughesdon in 1982, and the characteristics of ovarian fibrosis in PCOS were more collagenized tunica, thickened cortical and increased subcortical stroma [10]. Inhibiting ovarian fibrosis in PCOS can block the progression of PCOS, and thus it should be an effective strategy for PCOS treatment [11].

Previous studies have demonstrated that TGF-β1/Smads signal pathway plays a prominent part in the pathogenesis of organ fibrosis [11–13]. TGF-β1, a profibrotic cytokine, facilitates the excessive synthesis and deposition of extracellular matrix (ECM) and thus causes multiple organ fibrosis [9]. Fibrosis is the abnormal deposition of the ECM components in related tissues [14]. TGF-β/Smad signaling pathway is evoked when TGF-β1 binds to a transmembrane kinase, transforming growth factor β receptor II (TGFβRII) [15]. TGFβRII phosphorylates transforming growth factor β receptor I (TGFβRI), and then the cytoplasmic downstream mediators, which are Smad2 and Smad3, are phosphorylated by phosphorylated TGFβRI [16]. Phosphorylation of Smad2 and Smad3 forms a complex with the common mediator Smad4 which can be transferred into nucleus to regulate downstream proteins [15]. Smad7, a negative regulator of TGF-β1/Smads pathway, antagonizes TGF-β1-mediated fibrosis [17]. Many downstream proteins in the TGF-β1/Smads signal pathway including connective tissue growth factor, matrix metalloproteinases (MMPs), α-smooth muscle actin (α-SMA), participate in organ fibrosis. As a marker of active fibroblasts, α-SMA participates in the synthesis of various ECM components, including collagen I and collagen III, which regulate fibrosis in different tissues [18, 19]. MMPs are a family of proteolytic enzymes containing metal ions such as Ca^2+^ and Zn^2+^ in the structure, and there is certain degree of substrate specificity among MMPs [20]. The same kind of MMPs can degrade multiple EMC, and a certain EMC can be degraded by multiple MMPs with different degradation efficiencies by various enzymes [20].

Paeoniflorin (PAE) is one of the primary active ingredients of *Paeonia lactiflora Pall*, a traditional Chinese herbal medicine, which possesses multiple biological potentials, including impeding the inflammatory response [21], preventing the occurrence of cancers [22], improving cardiac remodeling [23], attenuating insulin resistance [24], and alleviating neuropathic pain [25]. Recently, PAE has also been reported to own an anti-fibrotic activity, including down-regulation of Smad3/4 and up-regulation of Smad7 expression in radiation-induced hepatic fibrosis model [12], inhibiting the early stages of TGF-β mediated epithelial-mesenchymal transition in alveolar epithelial cells [13], and attenuating cardiac fibrosis [23].

The current studies on the effects of PAE on anti-fibrosis mainly focus on hepatic fibrosis, pulmonary fibrosis and cardiac fibrosis, but its effects on ovarian fibrosis has not been defined. In this study, the role of PAE in the treatment of ovarian fibrosis and the underlying mechanism were comprehensively explored in a rat model of PCOS induced by DHEA. Our findings revealed that PAE ameliorated the pathological changes of PCOS, especially in ovarian fibrosis. Thus, we hold that PAE has potential therapeutic effects on PCOS.

## RESULTS

### Effects of PAE on body weight and ovary weight in PCOS rats

**Figure 1:**
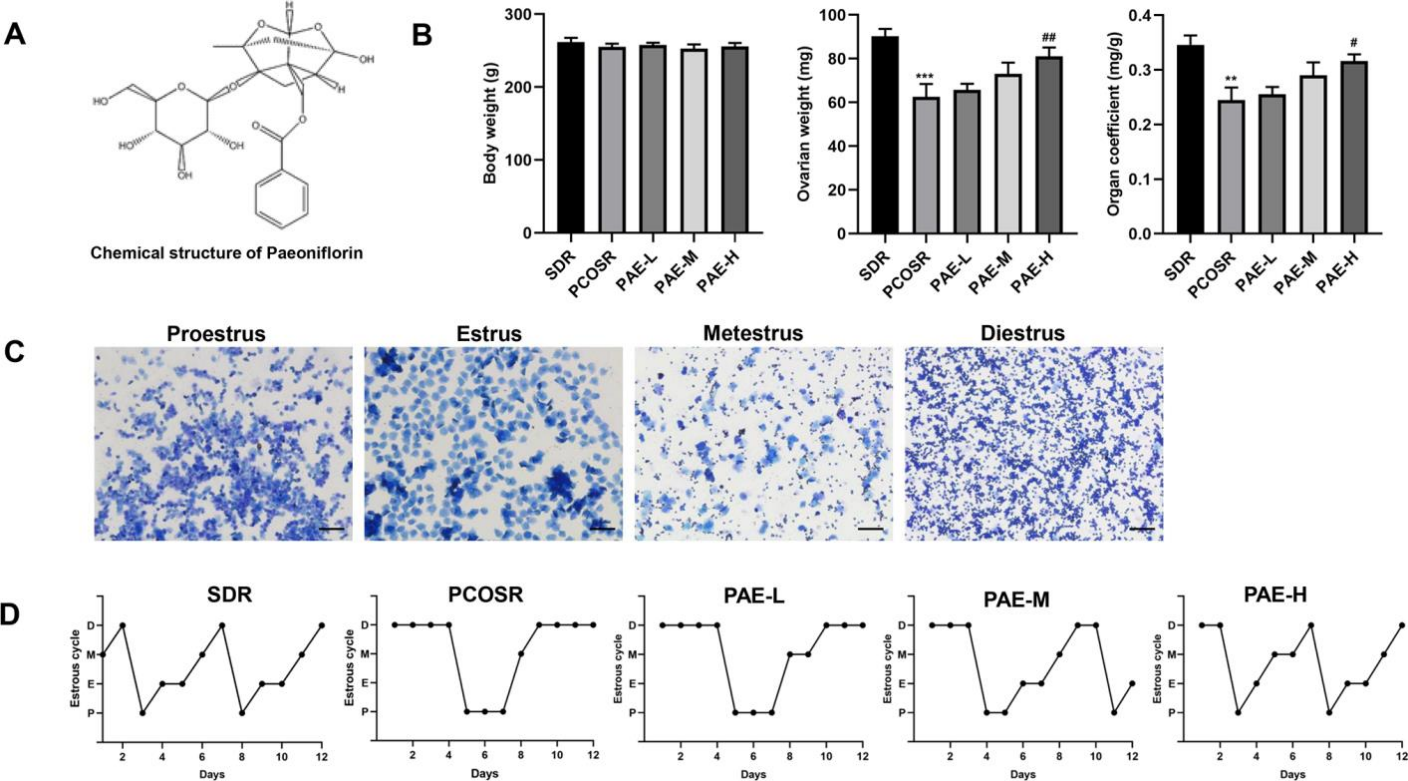
**Influence of PAE on body weight, ovary weight, organ coefficient and estrous cycle in PCOS rats (n=10 for each group).** (**A**) The chemical structure of PAE. (**B**) Influence of PAE on body weight, ovary weight and organ coefficient in PCOS rats (mean ± SEM, n=10 for each group). *P < 0.05, **P < 0.01 and ***P < 0.001 vs. SDR group. #*P* < 0.05, ##*P*< 0.01 and ###*P*< 0.01 vs. PCOSR group. (**C**) Different periods of estrous cycle (toluidine blue staining; Scale bars = 200 μm): Proestrus, Estrus, Metestrus and Diestrus. (**D**) Representative pictures of estrous cycle in five different groups; P, Proestrus; E, Estrus; M, Metestrus; D, Diestrus. PCOS, polycystic ovarian syndrome; SDR, normal control group; PCOSR, PCOS model group; PAE-L, PAE low-dose group (20 mg/kg/d); PAE-M, PAE middle-dose group (40 mg/kg/d); PAE-H, PAE high-dose group (80 mg/kg/d).

To explore the effect of PAE on PCOS development, the body weight and ovary weight in PCOS rats were monitored. We found that there was no statistically significant difference on the body weights in the five groups. In comparison with SDR group, the ovary weight and the organ coefficient of the PCOSR group were markedly reduced. The treatment of high-dose PAE (Figure 1A) could significantly block the DHEA-induced decline of ovary weight and organ coefficient (P < 0.05) (Figure 1B). These data suggested that PAE had potential therapeutic effect on PCOS.

### Effects of PAE on estrous cycle in rats with PCOS

To further confirm the effect of PAE on PCOS development, estrous cycle was detected by toluidine blue staining. The regular estrous cycle consists of four continuous periods including proestrus (P), estrus (E), metestrus (M) and diestrus (D), determined by the three kinds of vaginal epithelium cells (cornified cells, nucleated cells and leukocyte cells) [26]. The rats in PCOSR group lost the regular estrous cycle, most of which stayed in the diestrus period with the presence of a great number of leukocyte cells. By contrast, the rats in SDR group exhibited regular estrous cycle. And treatment of PAE could shorten the prolonged diestrus period and correct the irregular estrous cycle (Figure 1C, 1D), suggesting PAE played a regulative role in the estrous cycle of PCOS rats.

### Effects of PAE on ovarian structure and follicle growth in PCOS rats

**Figure 2:**
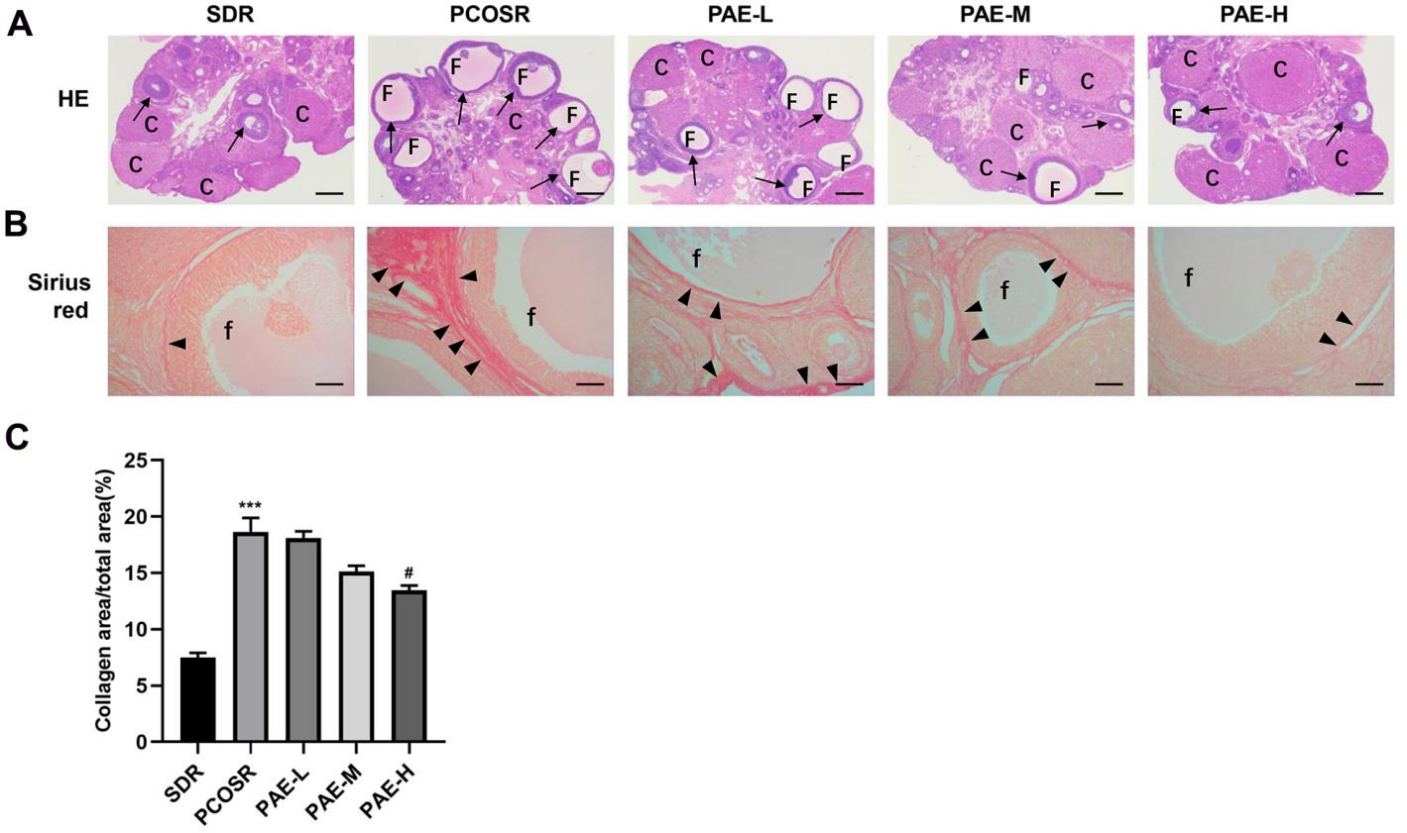
**Influence of PAE on ovarian structure, follicle growth and ovarian collagen deposition in PCOS rats.** Rats were executed at diestrus. (**A**) Representative pictures of ovarian tissues in PCOS rats (Hematoxylin and eosin staining, Scale bars = 500 μm, n=10 for each group). (**B**) Sirius red staining under a light microscope (Scale bars = 100 μm, n=10 for each group). (**C**) Proportion of the area containing collagen to the ovarian area (mean ± SEM, n=10 for each group). *P < 0.05, **P < 0.01 and ***P < 0.001 vs. SDR group. #P < 0.05, ##P< 0.01 and ###P< 0.01 vs. PCOSR group. PCOS, polycystic ovarian syndrome; SDR, normal control group; PCOSR, PCOS model group; PAE-L, PAE low-dose group (20 mg/kg/d); PAE-M, PAE middle-dose group (40 mg/kg/d); PAE-H, PAE high-dose group (80 mg/kg/d). “▲” is directed in the direction of collagen fibers. “→” is directed in the direction of granular cell layers. C, corpora luteum; F, cyst-like follicles; f, cyst-like follicles.

To further study the effect of PAE on PCOS development, the ovary structure was analyzed by H&E staining. In SDR group, multiple corpus luteum and follicles in different stages were observed from ovarian tissue slices by the microscope, and cystic dilated follicles were rarely found. Whereas in PCOSR group, the ovaries showed polycystic changes with cystic dilated follicles. The number of corpus luteum was significantly reduced, and the granular cell layer in the follicles was also reduced compared with SDR group. The above pathological changes could be effectively improved after the treatment with PAE. Compared with PCOSR group, the number of cystic dilated follicles in the ovarian was tissues decreased, while the number of corpus luteum was increased, and the granular cell layers were thickened in the ovaries in PAE-M and PAE-H groups. The improvement was most obvious in the PAE-H group (Figure 2A). These results indicated that PAE ameliorated the pathological symptoms of the ovary.

### Effects of PAE on collagen deposition in PCOS rats

Sirius red staining (Figure 2B) revealed that only a few collagen fibers were present in the ovarian tissues in SDR group. In contrast, more collagen fibers were clearly observed in PCOSR group. Treatment with high-dose PAE markedly reduced ovarian fibrosis (P < 0.05), which indicated that PAE improved ovarian fibrosis and collagen deposition caused by DHEA. The analysis of the collagen areas also presented that PAE markedly reduced the collagen areas (Figure 2C).

### Effects of PAE on the reproductive hormone levels in PCOS rats

**Figure 3:**
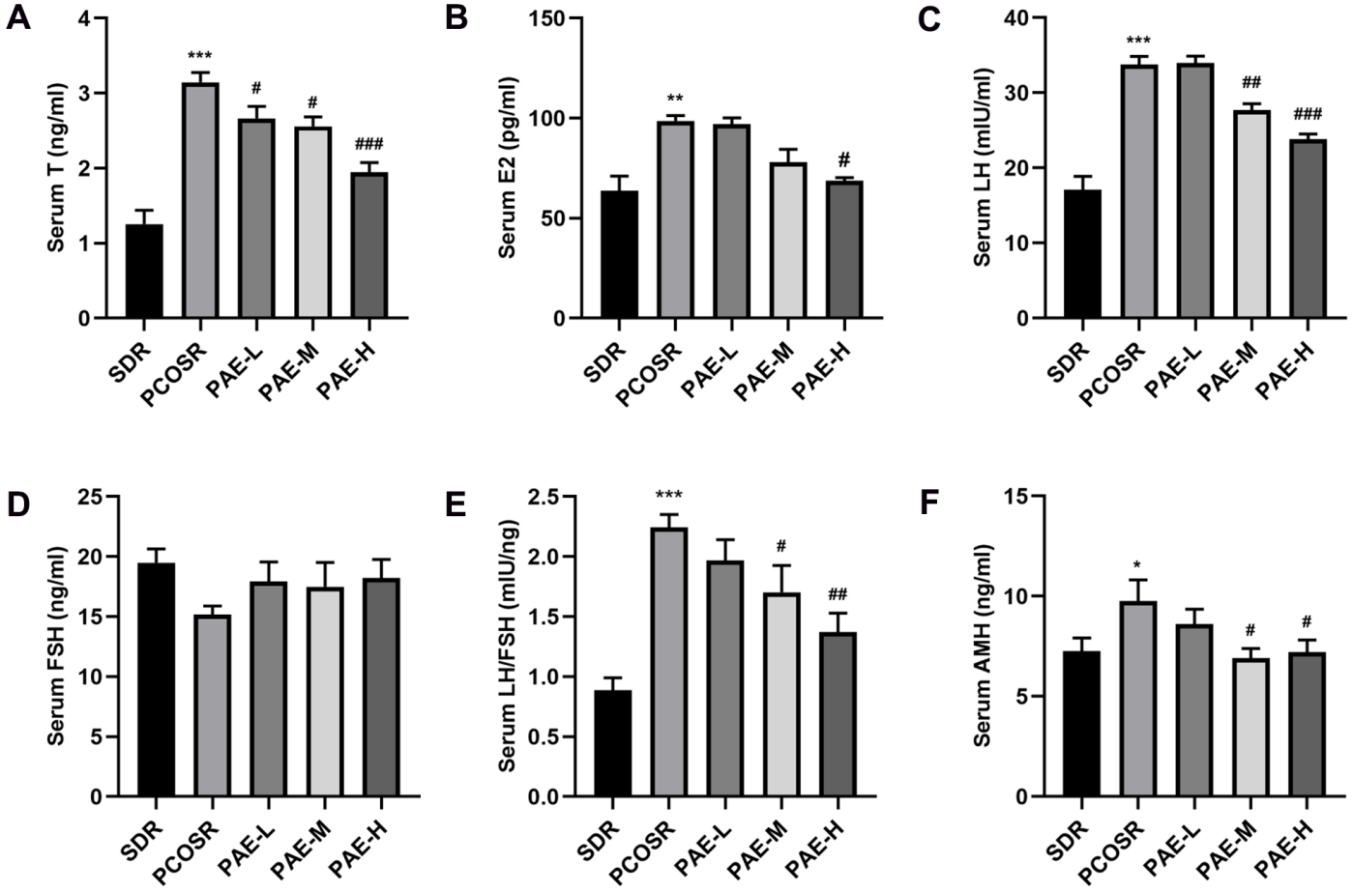
**Influence of PAE on the reproductive hormone levels in PCOS rats (mean ± SEM, n=10 for each group).** (**A**–**D**, **F**) The levels of serum T, E2, LH, FSH and AMH. *P < 0.05, **P < 0.01 and ***P < 0.001 vs. SDR group. #*P* < 0.05, ##*P*< 0.01 and ###*P*< 0.01 vs. PCOSR group. (**E**) The ratio of LH to FSH. *P < 0.05, **P < 0.01 and ***P < 0.001 vs. SDR group. #*P* < 0.05, ##*P*< 0.01 and ###*P*< 0.01 vs. PCOSR group. PCOS, polycystic ovarian syndrome; SDR, normal control group; PCOSR, PCOS model group; PAE-L, PAE low-dose group (20 mg/kg/d); PAE-M, PAE middle-dose group (40 mg/kg/d); PAE-H, PAE high-dose group (80 mg/kg/d); T, total testosterone; E2, estradiol; LH, luteinizing hormone; FSH, follicle stimulating hormone; AMH, Anti-Mullerian Hormone.

To further study the effect of PAE on PCOS development, the reproductive hormone levels were determined by ELISA. Compared with SDR group, the serum levels of T, LH, and LH/FSH were markedly increased in PCOSR group (P <0.001). In PCOSR group, the serum levels of E2 (P < 0.01) and AMH (P < 0.05) showed statistical significance, whereas no statistical difference was noted in FSH (P > 0.05). In contrast, the serum levels of T, E2, LH, LH/FSH and AMH were significantly reduced in the PAE group compared with PCOSR group (P <0.05), while the serum level of FSH showed no statistical difference (P >0.05). The occurrence of hyperandrogenism and excessive LH secretion are important features of the pathophysiology of PCOS [19], indicating the PCOS model was successfully constructed in the present study. Treatment of PAE could significantly reduce the serum levels of T, LH, E2, AMH and LH/FSH in PCOS rats, which displayed that PAE could improve the reproductive hormone levels of PCOS (Figure 3A–3F). These results implied that PAE may have corrective effect on reproductive endocrine of female.

### Effect of PAE on the activation of TGF-β1/Smads signaling pathway in ovarian tissues of PCOS rats

**Figure 4:**
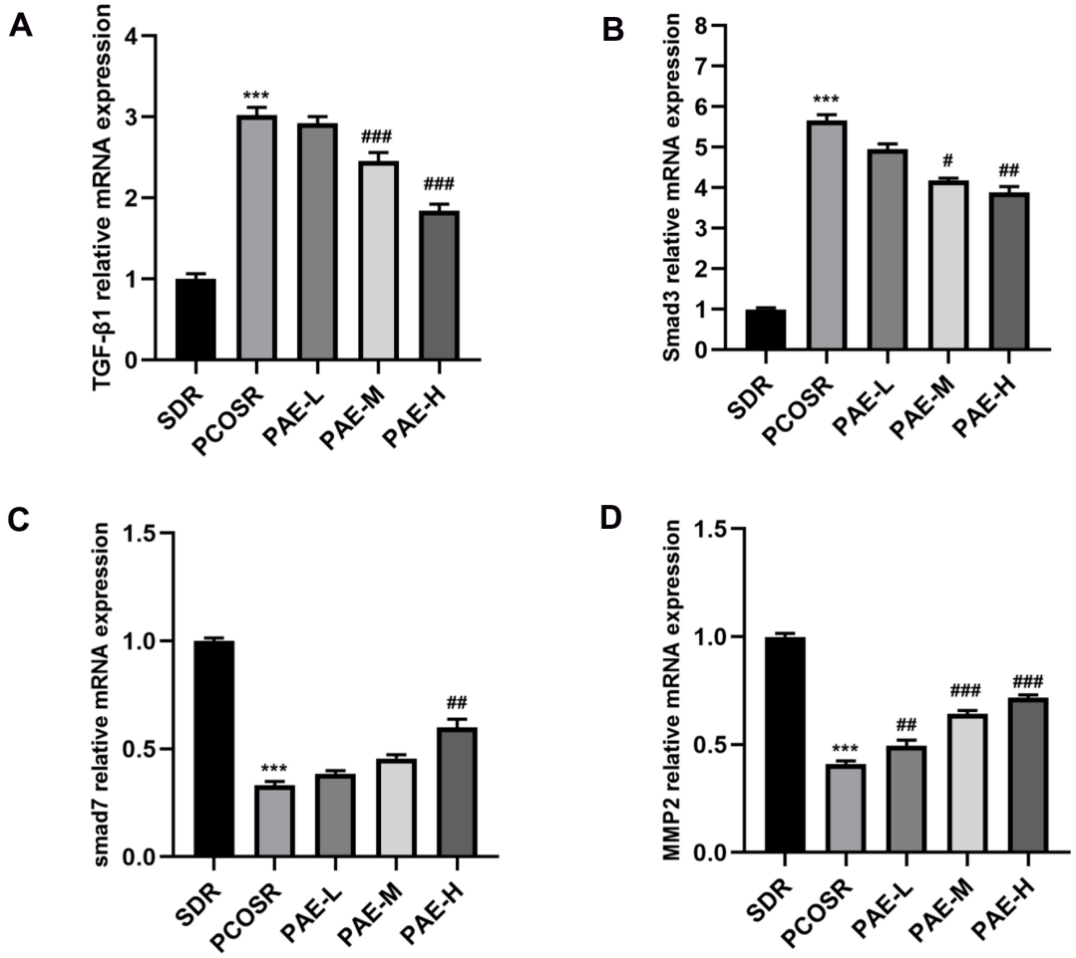
**Influence of PAE on the activation of TGF-β1/Smads signaling pathway in PCOS rats was evaluated by qRT-PCR (mean ± SEM, n=10 for each group).** (**A**, **B**) The mRNA levels of TGF-β1 and Smad3 in the ovarian tissues of PCOSR group were significantly higher than those in the SDR group (P < 0.001). The treatment with PAE reduced the mRNA levels of TGF-β1 and Smad3 (P < 0.05). *P < 0.05, **P < 0.01 and ***P < 0.001 vs. SDR group. #*P* < 0.05, ##*P*< 0.01 and ###*P*< 0.01 vs. PCOSR group. (**C**, **D**) The mRNA expression levels of Smad7 and MMP2 in the ovarian tissues of PCOSR group were significantly lower than those in the SDR group (P < 0.001). The Treatment with PAE increased the mRNA expression levels of Smad7 and MMP2 (P < 0.05). *P < 0.05, **P < 0.01 and ***P < 0.001 vs. SDR group. #*P* < 0.05, ##*P*< 0.01 and ###*P*< 0.01 vs. PCOSR group. PCOS, polycystic ovarian syndrome; SDR, normal control group; PCOSR, PCOS model group; PAE-L, PAE low-dose group (20mg/kg/d); PAE-M, PAE middle-dose group (40mg/kg/d); PAE-H, PAE high-dose group (80mg/kg/d).

To investigate the specific mechanism underlying the effect of PAE on PCOS development, the TGF-β1/Smads signaling pathway was determined via qRT-PCR and western blot. The mRNA expression levels of TGF-β1 and Smad3 in the ovarian tissues of PCOSR group were significantly higher than those in SDR group (P < 0.001), and the mRNA expression levels of Smad7 and MMP2 in the ovarian tissues of PCOSR group were significantly lower than those in SDR group (P < 0.001). Treatment with PAE reduced the relative mRNA levels of TGF-β1 and Smad3 (P < 0.05) and increased the mRNA levels of Smad7 and MMP2 (P < 0.05) (Figure 4A–4D).

**Figure 5:**
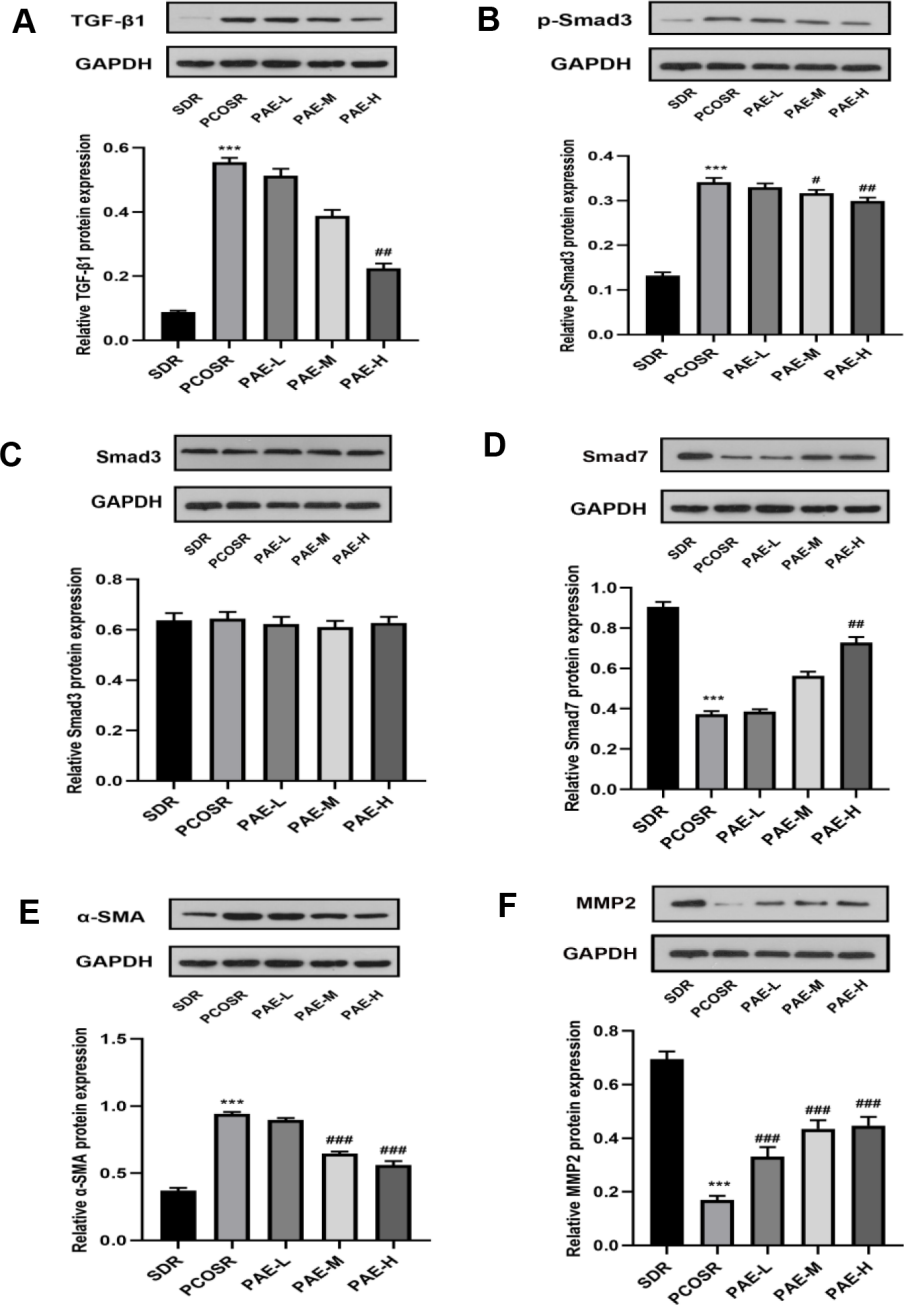
**Influence of PAE on the activation of TGF-β1/Smads signaling pathway in PCOS rats was evaluated by western blot.** The protein expressions of (**A**) TGF-β1, (**B**) p-Smad3, (**C**) Smad3, (**D**) Smad7, (**E**) α-SMA and (**F**) MMP2 were detected in ovarian tissues of PCOS rats. Same one GAPDH was used as the internal standard in (**A**, **D**, **E**). Same one GAPDH was used as the internal standard in (**B**, **C**, **F**). *P < 0.05, **P < 0.01 and ***P < 0.001 vs. SDR group. #P < 0.05, ##P< 0.01 and ###P< 0.01 vs. PCOSR group. PCOS, polycystic ovarian syndrome; SDR, normal control group; PCOSR, PCOS model group; PAE-L, PAE low-dose group (20 mg/kg/d); PAE-M, PAE middle-dose group (40 mg/kg/d); PAE-H, PAE high-dose group (80 mg/kg/d).

The protein expression levels of TGF-β1, p-Smad3 and α-SMA in the ovarian tissues of PCOSR group were significantly higher than those in the SDR group(P < 0.001), and the protein expression levels of Smad7 and MMP2 in the ovarian tissues of PCOSR group were significantly lower than those in SDR group (P < 0.001). There was no statistically significant difference in the protein expression level of Smad3. Treatment with PAE corrected the reduction in protein expression levels of Smad7 and MMP2 (P < 0.05), and reduced the protein expression levels of TGF-β1, p-Smad3 and α-SMA (P < 0.05) (Figure 5A–5F). These results suggested that PAE attenuated DHEA-induced polycystic ovary syndrome via inhibiting TGF-β1/Smads signaling pathway in PCOS rats.

## DISCUSSION

The purpose of this study was to clarify the therapeutic effect of PAE on ovarian fibrosis in DHEA-induced PCOS rats and explore whether PAE could exert anti-fibrotic effects on the ovary by inhibiting the TGF-β1/Smads signaling pathway.

The DHEA-induced PCOS model successfully generated the phenotypes, including disturbed estrous cycle, increased numbers of atretic follicles and follicles with cystic dilatation, which were consistent with the previous findings [19, 26]. In this study, PCOS rats had higher levels of serum T, LH, and AMH, which might be related to PCOS induced by DHEA. Hyperandrogenism is the diagnostic component of PCOS, with free testosterone being the most sensitive marker of androgens [27]. This study proved that PAE could reduce serum levels of T, LH, and AMH. The reduction in T level was likely to play a crucial role in the improvement of endocrine function in PCOS rats, which was closely related to the following mechanisms: (i) TGF-β1 was abnormally increased in PCOS patients, playing an important part in the pathophysiology of PCOS [28]. This finding indicated that the high expression of TGF-β1 might be related to the occurrence of hyperandrogenism in PCOS. Our research proved that PAE could attenuate ovarian tissue fibrosis and reduce androgen levels by inhibiting expression of TGF-β1; (ii) CYP17A1 and CYP11A1 are recognized as major enzymes of the androgen metabolic pathways [29]. The inhibition of CYP17A1 suppressed intratumoral steroid synthesis [30]. PAE could reduce the excessive testosterone in theca cells through downregulation of CYP17A1 and CYP11A1, which provided scientific evidence for the treatment of ovarian hyperandrogenism with PAE [31]. Insulin resistance and hyperandrogenism are found in more than 50% women with PCOS, which are independent of obesity [32].

Elevated insulin levels can directly stimulate the production of ovarian androgen, and elevated androgen levels can cause menstrual disorders, hirsutism, ovarian cysts, ovarian fibrosis and other PCOS-related diseases [19, 33]. Myo-inositol (MI) and D-chiro-inositol (DCI), known as insulin sensitizers, are of proven utility in the treatment of PCOS [34, 35]. MI is a second messenger of follicle-stimulating hormone (FSH) and DCI is an aromatase inhibitor in the human ovary [35]. Previous research found that PAE significantly decreased serum insulin and glucagon levels, improved insulin sensitivity and serum lipids profiles, and alleviated hepatic steatosis in Sprague-Dawley rats [36]. Therefore, we speculated that one of the possible mechanisms by which PAE improved ovarian fibrosis should be related to the reduction in androgen levels and inhibition of TGF-β1/Smads signaling pathway by improving insulin resistance.

Ovarian fibrosis is chiefly induced by ovarian injury which is caused by a lot of factors. Many cytokines can promote ECM deposition and the formation of fibrosis [9]. Our findings provided evidence that DHEA could facilitate the ovarian fibrosis in PCOS model rats (Figure 2B, 2C). The number of corpus luteum was significantly reduced, with granule cell layer of ovarian follicles in PCOSR group rats being thinner (Figure 2A). Therefore, ovarian fibrosis is related to the degeneration of granulosa cells and the deposition of collagen. TGF-β1 is considered as a crucial mediator in tissue fibrosis by activating its downstream small mother against decapentaplegic Smad signaling [17]. Previous studies on PAE have shown that PAE could ameliorate the organ fibrosis by inhibiting the TGF-β1/Smads signal pathway [12, 13, 37], while there is no research on the anti-fibrotic effect of PAE on ovarian fibrosis. Upon activation by TGF-β1, Smad3 is phosphorylated and then form a complex via Smads signaling. This complex is mediated by receptors that can be internalized in clathrin-coated pits and regulated by receptors, and further activates the transcription of TGF-β/Smad target genes [17]. The rise of Smad3 and TGF-β1 mRNA levels and the rise of protein expressions of pSmad3 and TGF-β1 in PCOSR group were detected in our study, whereas total Smad3 had no significant change, suggesting TGF-β/Smads signaling pathway contributes to PCOS pathogenesis. We firstly found that PAE had therapeutic effects on PCOS model and explored the underlying mechanism of PAE in ovarian fibrosis. Our results showed that PAE could reduce the expression of TGF-β1 and p-Smad3, and increased the expression of Smad7, especially the high dose of PAE (Figure 5A–5D).

This study found that treatment with high dose of PAE markedly reduced ovarian fibrosis, which indicated that PAE improved ovarian fibrosis and collagen deposition caused by DHEA. The analysis of the collagen areas also presented that PAE markedly reduced the collagen areas. PAE could reduce the collagen areas in ovarian tissues (Figure 2C), which was consistent with the results by the previous research on the effects of PAE on the treatment of liver fibrosis, myocardial fibrosis, and lung fibrosis [12, 13, 23, 37]. The different effects of PAE in reducing the deposition of collagen fibers may be related to the dosage of the drug, the method of drug administration and the time of drug treatment.

Accumulating evidence now suggests that the anti-fibrotic effect of PAE is still associated with the regulation of MAPK signal pathway, oxidative stress and inflammation [23, 31, 38]. Whether the inflammation can facilitate the occurrence of ovarian fibrosis and whether PAE can revise ovarian fibrosis by inhibiting inflammation with the crosstalk of TGF-β1/Smads signaling pathway and MAPK signaling pathway remain to be studied. Consequently, we plan to explore the effect of PAE on ovarian fibrosis upon MAPK signal pathway *in vivo* and *in vitro*.

Although there is a great number of research on fibrosis, the pathological mechanism of ovarian fibrosis is still unclear. Our findings have confirmed that ovarian fibrosis in PCOS is closely related to TGF-β1/Smads signaling pathway. Moreover, PAE can be used in the treatment of PCOS ovarian fibrosis, and its therapeutic effect may be due to its regulation on TGF-β1/Smads signaling pathway and the effects of its downstream signaling molecules.

## CONCLUSIONS

PAE has great prospects in the treatment of ovarian fibrosis in PCOS rats by regulating TGF-β1/Smads pathway. It is necessary for us to further explore the mechanism of PAE in improving PCOS ovarian fibrosis and to translate PAE into the treatment of clinical PCOS patients.

## MATERIALS AND METHODS

### Animals

Fifty-eight 3-week-old female Sprague-Dawley (SD, 40-60 g) rats were supplied by the Animal Care of Nanjing Medical University (Nanjing, China). The experimental approach was overseen and approved by the Ethical Committee of Nanjing Medical University. All of the animals were kept in the plastic cages under specific pathogen-free (SPF) grade on a 12-hour light/dark cycle with a temperature of 20-24° C and humidity of 50% and given *ad libitum* access to sterilized food and water. Beddings of these animals were changed twice a week.

### Experimental design and drug administration

Forty-eight SD rats (PCOS group, n=48) were given subcutaneous injection of 60 mg/kg/d dehydroepiandrosterone (DHEA; APExBIO Technology LLC, Houston, USA) diluted with 0.2 ml soybean for 35 consecutive days [19, 26]. The remaining rats (SDR group, n=10) were subcutaneously injected by 0.2 ml soybean oil (Shanghai Yuanye Biological Technology Co., LTD, Shanghai, China) per day. PCOS rats model was successfully established when irregular estrus cycle happened on rats. Ten PCOS rats were treated as PCOSR group (n=10), and thirty PCOS rats were divided into three groups (n=10) being administrated by gavage with Paeoniflorin (Chengdu Herbpurify Co., Ltd, Chengdu, China) on 20 mg/kg/d, 40 mg/kg/d, 80 mg/kg/d respectively [12]. Rats in SDR and PCOSR groups were treated with the same weight-based volume of distilled water by gavage for 4 weeks. All animals were euthanized under ether narcotization, and the specimens were collected for the following examinations.

### Body weight, ovary weight and estrous cycles

The body weights of the rats were monitored daily, and the rat ovaries were weighed after anesthetization. Estrous cycle, constituted by proestrus (P), estrus (E), metestrus (M) and diestrus (D), was determined by three kinds of vaginal epithelium cells. Vaginal smears were daily operated between 9:00 to 10:00 A.M. from the 14^th^ day to the end of the study. The vaginal secretion was smeared on the glass slides, and then the slides were dyed by toluidine blue (Beijing Solarbio Science and Technology Co., Ltd, Beijing, China) for 15 min.

### Blood sample analysis

Blood samples were collected from the blood in the abdominal aorta after anesthetization. After centrifugation at 3000 r/min for 10 min, the supernatants were collected and preserved for the measurements of serum testosterone (T), estradiol (E2), follicle stimulating hormone (FSH), luteinizing hormone (LH), and anti-mullerian hormone (AMH) according to the manufacturer’s protocols of the ELISA kits (Elabscience Biotechnology Co., Ltd, Wuhan, China).

### Ovarian haematoxylin and eosin staining and sirius red staining

The ovarian specimens were fixed in 4% paraformaldehyde after being harvested from rats, and then were embedded in paraffin. Ovarian paraffins were sliced at 4 μm thickness. Ovarian paraffin slices were stained with hematoxylin and eosin to observe the pathological changes of ovarian structure and morphology.

### Measurement of the collagen area

Ovarian paraffin slices were stained with Sirius-red staining to determine the collagen fibers in the ovarian tissues. The collagen area was calculated by Image J software.

### Quantitative real-time PCR (qRT-PCR) analysis

qRT-PCR analysis was used to analyze the mRNA levels of TGF-β1, Smad3, Smad7, and MMP2. Total RNA was extracted from ovarian tissues using Trizol reagent (Servicebio Biotechnology Co., Ltd., Wuhan, China). RNA concentration and purity were measured with a nucleic acid protein detector (Applied Biosystems, Carlsbad, USA). Subsequently, RNA (1μl) was reversely transcribed into cDNA according to the instructions of Reverse Transcription Kit (Thermo Fisher Scientific, Waltham, USA). PCR reactions were performed with 12.5 μL SYBR Premix (2x), 5 μL of PCR reverse primer, 5 μL of PCR reverse primer, 1.5 μL of cDNA and 1 μL ddH2O. The primer sequences of the target genes were compounded by Servicebio Biotechnology Co., Ltd and were presented as follows: TGF-β1 forward, 5'-CAACAATTCCTGGCGTTACCT-3' and reverse, 5'-GCCCTGTATTCCGTCTCCTT-3'; Smad3 forward, 5'- GGAATGCAGCCGTGGAACTT-3' and reverse, 5'-TTGCAGCCTGGTGGGATCTT-3'; Smad7 forward, 5'-CTCCTCCTTACTCCAGATACCCAA-3' and reverse 5'-TCTCCTCCCAGTATGCCACCA-3'; MMP2 forward, 5'- ACACCAAGAACTTCCGACTATCC -3' and reverse, 5'- GAGCAATGCCATCAAAGACAA-3'; GAPDH forward, 5'-TCTCTGCTCCTCCCTGTTC-3' and reverse, 5'-ACACCGACCTTCACCATCT-3'. The expressions of the target genes were normalized to GAPDH gene employing the 2^−ΔΔCT^ method.

### Western blotting

Total ovarian proteins were extracted by using RIPA lysis buffer containing protease inhibitors and protein phosphatase inhibitors (Servicebio Biotechnology Co., Ltd., Wuhan, China). The mixtures were homogenized and stored on ice for 10min, and then centrifuged at 12,000 rpm for 10 min at 4° C. The supernatant was used to conduct western blot analysis. The proteins (30 μg) were separated with 10% SDS-polyacrylamide gels and then transferred onto PVDF membranes (Millipore, Burlington, USA). After being blocked with 5% skimmed milk for 1 h, the strips of target proteins and internal protein were washed with TBST. The strips were each incubated with primary antibodies against TGF-β1, p-Smad3, Smad3, Smad7, MMP2, and GAPDH purchased from Servicebio Biotechnology Company (Wuhan, China) overnight at 4° C. The strips were then incubated with HRP-conjugated anti-rabbit IgG (CST, Danvers, USA) secondary antibodies for 2h at room temperature. The contents of target proteins were detected with an enhanced chemiluminescence agent (Tanon, Shanghai, China). The grey values of the protein strips were analyzed with Image J software (National Institutes of Health, Bethesda, USA). The expressions of target proteins were normalized to the protein of GAPDH.

### Statistical analysis

The measurement data were all expressed as the means ± standard error and analyzed with SPSS 22.0 statistical software. Multi-group comparisons were conducted using ANOVA followed by Dunnett post hoc test at the 0.05 level. KruskalWallis test was used when variables did not meet a normal distribution or homogeneity of variance. P <0.05 was considered as statistically significant.
